# Obstructive sleep apnea predicts pathologic response to neoadjuvant therapy in resected pancreatic ductal adenocarcinoma

**DOI:** 10.1002/mco2.184

**Published:** 2022-11-11

**Authors:** Sami Shoucair, Ning Pu, Joseph R. Habib, Elizabeth Thompson, Christopher Shubert, Richard A. Burkhart, William R. Burns, Jin He, Kelly J. Lafaro, Jun Yu

**Affiliations:** ^1^ Departments of Surgery and Pathology Johns Hopkins University School of Medicine Baltimore Maryland USA; ^2^ Department of Surgery MedStar Health Baltimore Maryland USA; ^3^ Departments of General Surgery Zhongshan Hospital Fudan University Shanghai China

Dear Editor:

Pancreatic ductal adenocarcinoma (PDAC) is one of the most lethal malignancies, with a 5‐year overall survival (OS) rate of 11% in the United States.^1^ Improved outcomes for localized PDAC are mainly attributed to the use of more potent combination chemotherapy regimens. Surgery, however, remains the only curative option. A growing body of evidence suggests that neoadjuvant therapy (NAT) improves surgery candidacy as well as postoperative outcomes in these patients, particularly in patients with a favorable pathologic response (PR).^2^ There is, however, a lack of predictors of response to therapy before surgical resection.

Obstructive sleep apnea (OSA) is a known prognostic factor that impacts the outcome of cancer patients. Approximately 20% of Americans are estimated to suffer from OSA, which represents a substantial public health burden.^3^ This disorder is characterized by repetitive upper airway obstruction during sleep, resulting in fragmented sleep and oxygen depletion. In addition, the resultant intermittent hypoxia triggers sympathetic activation and systemic inflammation. Consequently, pro‐inflammatory molecules are secreted more readily, and free radicals are produced, thereby creating an oxidative stress environment. Ultimately, this dysregulation leads to organ injury and an increased risk of cardiovascular and cerebrovascular disease morbidity and mortality.

Several cancer types have been associated with OSA as a potential risk factor for carcinogenesis and cancer‐related mortality, including colorectal, lung, and prostate cancer. OSA was found to be an independent predictor of lymph node metastasis in over 1000 patients with PDAC who underwent upfront surgical resection.^4^ To our knowledge, no study has examined the association of OSA with PDAC prior to resection in patients with PDAC. OSA should be investigated in these patients because hypoxia‐induced cellular and molecular dysregulation may interfere with remodeling the tumor microenvironment seen following NAT.

We conducted this study to investigate the association between OSA and cancer‐specific outcomes in patients who had undergone surgical resection of PDAC. Using the current tumor regression grading system according to the College of American Pathologists (CAP), favorable PR was defined as a CAP score of 0 or 1, and unfavorable PR as a CAP score of 2 or 3.^2^ In the analyzed cohort of patients who underwent NAT and resection for PDAC (*n* = 334) (Figure 1A and Table S1), OSA was significantly associated with male sex, resectable disease stage at diagnosis, shorter duration of NAT, increased tumor size, lymphovascular and perineural invasion, R1 margin, and unfavorable PR to NAT. In addition, OSA was an independent predictor of unfavorable PR in this study.

**FIGURE 1:**
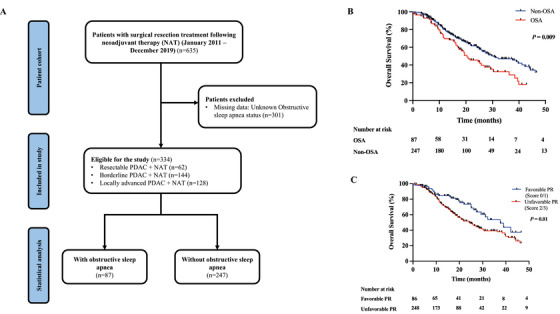
Flow diagram of study population (A), Kaplan–Meier survival curves comparing superior overall survival between obstructive sleep apnea (OSA) and non‐OSA patients (B), and between favorable PR (score 0/1) and unfavorable PR (score 2/3) (C). OSA, obstructive sleep apnea; non‐OSA, without obstructive sleep apnea; PR, pathologic response

According to univariable logistic regression (Table S2), OSA (odds ratio [OR], 3.01; 95% confidence interval [CI], 1.51–5.99; *p* = 0.002) was a significant predictor of an unfavorable PR, whereas White race (OR = 0.41; 95% CI, 0.18–0.90; *p* = 0.026), pre‐NAT BR/LAPC stage (OR, 0.49; 95% CI, 0.24–1.03; *p* = 0.059) and NAT with chemoradiation (OR, 0.36; 95% CI, 0.19–0.66) was associated with favorable PR. Multivariable analysis showed that OSA (OR, 2.72; 95% CI, 1.35–5.49; *p* = 0.005), White race (OR, 0.44; 95% CI, 0.19–0.99; *p* = 0.048), and NAT chemo‐radiation (OR, 0.39; 95% CI, 0.19–0.79; *p* = 0.008) were independently predictive of pathological response (Figure 1C and Figure S1B).

The median recurrence‐free survival (RFS) of the entire cohort was 13.4 months (95% CI, 11.5–15.3), and the 1‐ and 3‐year RFS rates were 54.9% and 25.1%, respectively, while the median OS was 27.3 months (95% CI, 23.6–31.0). The 1‐ and 3‐year OS rates were 77.5% and 42.0%, respectively. Among patients with OSA after NAT, the median survival was 12.6 months (95% CI, 11.1–14.1), which did not differ significantly from those without OSA (14.2 months [95% CI, 11.7–16.7], *p* = 0.091) (Figure S1A). It is noteworthy, however, that the median survival time for patients suffering from OSA was significantly shorter than for patients without OSA (20.4 months [95% CI, 14.3–26.5] vs. 30.5 months [95% CI, 21.4–39.6], *p* = 0.009). Patients with OSA had 1‐ and 3‐year OS rates of 68.4% and 28.9%, respectively, compared to 79.7% and 45.5% for those without OSA (Figure 1B, Tables S3 and S4 and Supporting Information Results).

We found that patients with OSA have larger tumors, lymphovascular invasion, and perineural invasion, all of which are associated with a more aggressive phenotype. The potential oncogenic mechanism of OSA may include activating signaling pathways such as hypoxia‐inducible factor 1‐alpha (HIF‐1α) and glycogen metabolism, promoting oxidative stress‐induced DNA damage and creating a pro‐inflammatory, immunosuppressive microenvironment. There may be a connection between intermittent hypoxia and oxidative stress and the promotion of malignant phenotypes.^5^ Martinez et al. found that HIF‐1α expression may be affected at the transcriptional level in OSA‐induced intermittent hypoxia and not simply through a posttranslational, oxygen‐dependent degradation pathway.^6^ Hypoxia intermittently has been shown to suppress ER‐alpha expression and diminish the response to endocrine therapy in breast cancer.

Nevertheless, no direct studies have been conducted on the effects of intermittent hypoxia on PDAC. As a consequence of high‐density fibro‐inflammatory stroma, tumors are hypoxic and their immune systems are suppressed, which alters the intratumorally metabolic pathways and results in a poor prognosis. Large amounts of hypoxia mechanisms on promoting PDAC cell progression have been illuminated, containing crosstalk between hypoxia‐sensing ULK1/2 and YAP‐driven glycolysis, HIF‐1α/Notch signaling pathway and noncoding RNA‐related signaling, etc.^7^, ^8^, ^9^ According to these studies, OSA may contribute to the aggressive phenotype of PDAC.

OSA may have an effect on PDAC prognosis after NAT since both have an impact on the cancer‐immune and metabolic environment. Our study found that OSA was an independent predictor of PR to NAT and was associated with worsened RFS and OS. Our research speculated that OSA could overcome the reversion of neoadjuvant therapy on cancer cells and the microenvironment and lead to a poor outcome. According to studies conducted on several other cancers, increasing evidence has been found that OSA‐induced hypoxia may be a promising indicator of unfavorable PR to NAT.

There are several limitations to be acknowledged. First, all patients with PDAC enrolled in the study underwent surgical resection after neoadjuvant treatment. PDAC patients who became or remained unresectable after neoadjuvant therapy were excluded from this study. As a result of the observational design of the study, almost half of the cases were excluded for lack of information regarding OSA. Even so, the presented series consists of a large cohort from a high‐volume surgical center. In order to validate the results of the analysis, a large‐scale, prospective, multicenter clinical trial is needed.

In conclusion, this study demonstrates that OSA can predict OS by correlation with pathologic response after neoadjuvant therapy in PDAC. OSA may be an auxiliary, robust, and promising predictive indicator in PDAC patients receiving neoadjuvant therapy, which may provide a novel therapeutic perspective to improve its response.

## AUTHOR CONTRIBUTIONS

*Conceived and designed the experiments*: Jun Yu and Sami Shoucair; *acquisition of data*: Sami Shoucair, Ning Pu, and Joseph R. Habib; *analysis and interpretation of data*: Sami Shoucair, Joseph R. Habib and Jun Yu; *drafted the manuscript*: Ning Pu, Sami Shoucair and Jun Yu; *revised the manuscript，  agreed with the manuscript's results and conclusions , and read and approved the final manuscript* : all the authors; *obtained funding*: Jun Yu and Ning Pu; and *study supervision*: Jun Yu.

## CONFLICT OF INTEREST

All authors declare no conflicts of interest.

## ETHICS STATEMENT

This study was approved by the JHH Institutional Review Board and performed in accordance with the Declaration of Helsinki.

## Supporting information



Supporting Information

## Data Availability

The data sets used and analyzed during the current study are available from the corresponding author upon reasonable request.
