# Supplementary material for: Obstructive sleep apnea predicts pathologic response to neoadjuvant therapy in resected pancreatic ductal adenocarcinoma
Source: MedComm (2020). 2022 Nov 11;3(4):e184. doi: 10.1002/mco2.184 (PMC9652137; doi:10.1002/mco2.184)

**Obstructive Sleep Apnea Predicts Pathologic Response to Neoadjuvant Therapy in Resected Pancreatic Ductal Adenocarcinoma**

*Sami Shoucair^1,2,*^ MD, Ning Pu^1,3,*^ MD, PhD, Joseph R. Habib^1^ MD, Elizabeth Thompson^4^ MD, PhD, Christopher Shubert^1^ MD, Richard A. Burkhart^1^ MD, William R. Burns^1^ MD, Jin He^1^ MD, PhD, Kelly J. Lafaro^1^ MD, PhD, and Jun Yu^1^ MD, PhD*

*Departments of ^1^Surgery and ^4^Pathology, Johns Hopkins University School of Medicine, Baltimore, Maryland, United States of America*

*^2^ Department of Surgery, MedStar Health, Baltimore, Maryland, United States of America*

*^3^Departments of General Surgery, Zhongshan Hospital, Fudan University, Shanghai, China*

* SS and NP contributed equally as co-first authors to the manuscript.

**Correspondence and requests for reprints to:**

Jun Yu, MD, PhD, Division of Hepatobiliary and Pancreatic Surgery, Johns Hopkins University. 600 N. Wolfe Street, Blalock 603, Baltimore, MD 21287. Email: jyu41@jhmi.edu

**Methods**

*Patient Cohort*

The institutional, prospectively maintained pancreas database at the Johns Hopkins Hospital was queried for surgically resected patients who received NAT between January 2011 and December 2019. Eligibility was determined by, i) a histological confirmation of PDAC, ii) a diagnosis of OSA prior to chemotherapy induction, iii) the availability of follow-up information (**Fig 1**). Patients were considered positive for OSA if a diagnosis of OSA was reported in the anesthesia records and/or elsewhere in the preoperative medical record. Information about current or past therapy with either continuous positive airway pressure (CPAP) or other devices to treat OSA was considered a surrogate indicator of OSA ([28](#_ENREF_28),[31](#_ENREF_31)). Patients with unknown prevalence of OSA were excluded from the analysis. Staging at time of diagnosis was evaluated by the JHH pancreatic multi-disciplinary clinic (PMDC) into resectable, borderline resectable, locally advanced or metastatic disease.

*Data Collection and Longitudinal Follow-Up*

Clinical, pathological and demographic data were obtained from the registry or, if missing, retrospectively collected from electronic medical records (EMR). All pathologic diagnoses at JHH were staged using the 8^th^ edition of the American Joint Committee on Cancer (AJCC) classification ([32](#_ENREF_32)). Using the current tumor regression grading system, PR to NAT was routinely determined by an independent pathologist (blinded to OSA assessment) through examination of resected primary tumor surgical specimens according to the College of American Pathologists (CAP) score ([7](#_ENREF_7),[8](#_ENREF_8),[10](#_ENREF_10),[13](#_ENREF_13),[33](#_ENREF_33)). Favorable PR was defined as a CAP score of 0 or 1, and unfavorable PR as a CAP score of 2 or 3 ([7](#_ENREF_7),[13](#_ENREF_13)). Overall survival (OS) was defined from the time of surgical resection to the date of death or censored as date of last follow-up. Survival status was collected using the Centers for Disease Control and Prevention National Death Index. December 31, 2020 was considered the date of the last follow-up unless subsequent visits were registered in the EMR. Recurrence-free survival (RFS) was defined from the time of surgical resection to recurrence as detected by routine imaging surveillance or cancer-related death, whichever occurred first.

*Statistical Analyses*

All statistical analyses were performed utilizing SPSS statistical package (version 28; IBM, NY). A chi-squared or Fisher's exact test and Mann-Whitney U test were utilized for univariable analysis for categorical and continuous variables, respectively. Continuous variables were displayed as medians with interquartile ranges (IQR). A logistic regression model was applied to identify factors independently associated with pathologic response. All time-to-event analyses (OS and RFS) were expressed as Kaplan-Meier curves and were compared using the log-rank test. Kaplan-Meier curves were truncated when less than 5% of the patients at risk remained. Kaplan-Meier curves were plotted utilizing Prism (version 9.3; GraphPad, CA). Risk-adjusted odd ratios (OR) with 95% confidence interval (CI) were employed using a logistic regression model for univariable and multivariable analysis. Risk-adjusted hazard ratios (HR) with 95% CI were employed using a proportional hazard Cox regression model for univariable and multivariable analysis. All factors with a value of *P* < 0.10 in univariable analysis were included in the multivariable analysis. A P value < 0.05 was used to determine statistical significance.

**Results**

*Demographics and tumor characteristics*

According to the inclusion and exclusion criteria, 334 resected PDAC patients after neoadjuvant therapy were identified (**Fig 1** and **Table S1**). In this overall cohort, the median age was 65.9 years (IQR 59.1-70.9) and 168 patients (50.3%) were male. A diagnosis of OSA was prevalent in 26% of patients. The median duration of NAT was 4.8 (IQR 3.2-7.1) months, with 73.3% of patients receiving 5-FU-based NAT compared to 26.7% who received Gem-based NAT. In addition, 222 patients (66.4%) also received neoadjuvant chemo-radiation. Additional adjuvant chemotherapy was offered to 188 patients (56.8%).

Tumor histopathological evaluation revealed that 20 patients were ypT0 (5.9%), 101 were ypT1 (30.2%), 105 were ypT2 (31.4%), 87 were yPT3 (26%) and 21 were ypT4 (6.3%). Nodal stage was 57.8%, 37.7% and 4.5% for ypN0, ypN1 and ypN2 respectively. Moreover, a favorable PR was appreciated in 25.7% of samples. Additional clinicopathological details are summarized in **Table S1**.

*Correlation between obstructive sleep apnea and clinicopathological characteristics*

In **Table S1**, patients with OSA were more likely to be male (63.2%, *P* = 0.005), resectable PDAC (31.0%, *P*=0.002), received shorter NAT duration (median, 4.0 months; IQR, 2.9-6.5; *P* = 0.042), higher ypT-stage (*P*=0.031), had lymphovascular invasion (48.3%, *P*=0.012), perineural invasion (73.6%, *P*=0.002), an R1 margin (18.4%, *P*=0.003), and poorer pathologic response to NAT (87.4%, *P*=0.001). No significant relationship was found between OSA and pre-NAT baseline Ca19-9 level, ypN-stage, tumor differentiation, chemo-radiation therapy and radiation type, etc.

*Prognostic significance of obstructive sleep apnea*

In addition, the median OS of patients with favorable PR was prolonged compared to those with unfavorable PR (86 vs. 248 patients, 37.6 months [95% CI, 28.2-46.9] vs. 25.1 months [95% CI, 19.9-28.8], *P*=0.010) (**Fig 1**). The median RFS of patients with favorable PR was significantly elevated compared to those with unfavorable PR (23.8 months [95% CI, not reached 50% by the date of the last follow-up] vs. 12.5 months [95% CI, 10.8-14.2], *P*<0.001) (**Fig S1**). The 1- and 3-year RFS rates for patients with favorable PR were 64.5% and 45.7%, and for patients with unfavorable pathologic response were 50.7% and 18.7%, respectively. Furthermore, the 1- and 3-year OS rates for patients with favorable PR were 82.9% and 49.1%, and for patients with unfavorable pathologic response were 74.6% and 38.0%, respectively.

On univariable Cox regression, the significant predictors of poorer RFS were baseline Ca19-9 ≥ 200, poor grade of tumor differentiation, increased tumor size, positive nodal status (≥N1), OSA, presence of lymphovascular and perineural invasion. In contrast, white ancestry and favorable PR were significant predictors of improved RFS (**Table S4**). Risk-adjusted multivariable Cox regression analysis found that nodal status ≥N1 (HR, 1.54; 95% CI, 1.10-2.16; *P=*0.012) was an independent predictor of a worse RFS. Moreover, white ancestry (HR, 0.65; 95% CI, 0.44-0.94; *P=*0.022) was identified as an independent predictor of an improved RFS on multivariable regression modeling. There was no significant difference in RFS based on age (≥65 years old), male, ASA (≥3), pre-NAT stage (BR/LAPC vs. resectable PDAC), NAT regimen (5-FU-based vs. Gemcitabine-based), durations of NAT, NAT chemoradiotherapy (vs. chemotherapy alone), R1 margin, or receiving adjuvant chemotherapy as univariable predictors.

On the univariable Cox regression analysis for OS, favorable PR (HR, 0.57; 95% CI, 0.39-0.89; *P*=0.010) and administration of adjuvant chemotherapy (HR, 0.72; 95% CI, 0.52-0.99; *P*=0.045) were confirmed as protective factors (**Table S3**). However, treatment-naïve baseline Ca19-9 ≥200 (HR, 1.34; 95% CI, 0.96-1.86; *P*=0.085), poorly differentiated tumor grade (HR, 1.58; 95% CI, 1.13-2.22; *P*=0.008), ypT-stage ≥T3 (HR, 1.62; 95% CI, 1.17-2.24; *P*=0.004), ypN-stage ≥N1 (HR, 2.15; 95% CI, 1.55-2.97; *P*<0.001), lymphovascular invasion (HR, 2.34; 95% CI, 1.68-3.26; *P*<0.001), perineural invasion (HR, 2.24; 95% CI, 1.55-3.25; *P*<0.001) and OSA (HR, 1.58; 95% CI, 1.12-2.24; *P*=0.009) were risk factors for OS. There was no significant difference in OS based on age, male, white ancestry, ASA (≥3), pre-NAT stage (BR/LAPC vs resectable PDAC), NAT regimen (5-FU-based vs Gemcitabine-based), durations of NAT, NAT chemoradiotherapy (vs chemotherapy alone), or R1 margin as univariable predictors.

On multivariable Cox regression for OS, poorly differentiated tumor grade (HR, 1.60; 95% CI, 1.12-2.29; *P*=0.010), ypN-stage ≥N1 (HR, 1.89; 95% CI, 1.27-2.80; *P*=0.002), lymphovascular invasion (HR, 1.53; 95% CI, 1.05-2.26; *P*=0.028), favorable PR (HR, 0.64; 95% CI, 0.42-0.97; *P*=0.036) and administration of adjuvant chemotherapy (HR, 0.57; 95% CI, 0.39-0.81; *P*=0.002) were significant predictive indicators (**Table S3**).

**Table S1.** Demographics and tumor characteristics of overall cohort (N=334) and univariable analysis of cohort without OSA (N=247) and with OSA (N=87).

| **N (%)** | **Overall Cohort**  **(N=334)** | **No OSA**  **(N=247)** | **OSA**  **(N=87)** | ***P* Value** |
| --- | --- | --- | --- | --- |
| Age (years) Median (IQR) | 65.9 (59.2 – 71.0) | 65.6 (59.0 – 70.7) | 67.0 (59.7 – 72.2) | 0.293 |
| Gender (Male) | 168 (50.3) | 113 (45.7) | 55 (63.2) | **0.005** |
| Race (White) | 276 (82.6) | 208 (84.2) | 68 (78.2) | 0.200 |
| Pre-NAT Stage |  |  |  | **0.002** |
| Resectable | 62 (18.6) | 35 (14.2) | 27 (31.0) |  |
| Borderline resectable | 144 (43.1) | 115 (46.6) | 29 (33.3) |  |
| Locally advanced | 128 (38.3) | 97 (39.3) | 31 (35.6) |  |
| ASA ≥ 3 | 253 (80.8) | 188 (81.0) | 65 (80.2) | 0.877 |
| Type of Neoadjuvant |  |  |  | 0.105 |
| 5-FU-based | 244 (73.3) | 186 (75.6) | 58 (66.7) |  |
| Gem-based | 89 (26.7) | 60 (24.4) | 29 (33.3) |  |
| Duration of NAT (months) Median (IQR) | 4.8 (3.2 – 7.1) | 5.0 (3.3 – 7.1) | 4.0 (2.9 – 6.5) | **0.042** |
| Chemo-Radiation Therapy | 222 (66.5) | 171 (69.2) | 51 (58.6) | 0.071 |
| Radiation Type |  |  |  | 0.336 |
| SBRT | 184 (55.1) | 44 (84.2) | 40 (78.4) |  |
| Standard radiation | 38 (11.4) | 27 (15.8) | 11 (21.6) |  |
| Ca19-9 Median (IQR) | 131 (13.7 – 636.8) | 126 (14.0 – 601.0) | 136 (9.1 – 981.9) | 0.779 |
| Tumor location (Head/neck) | 248 (74.3) | 184 (74.5) | 64 (73.6) | 0.864 |
| ypT-stage |  |  |  | **0.031** |
| T0 | 20 (6.0) | 18 (7.3) | 2 (2.3) |  |
| T1 | 101 (30.2) | 83 (33.6) | 18 (20.7) |  |
| T2 | 105 (31.4) | 69 (27.9) | 36 (41.4) |  |
| T3 | 87 (26.0) | 63 (25.5) | 24 (27.6) |  |
| T4 | 21 (6.3) | 14 (5.7) | 7 (8.0) |  |
| ypN-stage |  |  |  | 0.170 |
| N0 | 193 (57.8) | 150 (60.7) | 43 (49.4) |  |
| N1 | 126 (37.7) | 86 (34.8) | 43 (46.0) |  |
| N2 | 15 (4.5) | 11 (4.5) | 4 (4.6) |  |
| Grade of differentiation |  |  |  | 0.383 |
| Unknown | 20 (6.0) | 18 (7.3) | 2 (2.3) |  |
| Well | 15 (4.8) | 11 (4.8) | 4 (4.7) |  |
| Moderate | 198 (63.1) | 146 (63.8) | 52 (61.2) |  |
| Poor | 101 (32.2) | 72 (31.4) | 29 (34.1) |  |
| Lymphovascular Invasion | 124 (37.1) | 82 (33.2) | 45 (48.3) | **0.012** |
| Perineural Invasion | 199 (59.6) | 135 (54.7) | 64 (73.6) | **0.002** |
| Margin Class (R1) | 34 (10.2) | 18 (7.3) | 16 (18.4) | **0.003** |
| Adjuvant Chemotherapy | 188 (56.8) | 138 (56.1) | 50 (58.8) | 0.662 |
| Pathologic Response (Score) |  |  |  | **<0.001** |
| 0 | 20 (6.0) | 18 (7.3) | 2 (2.3) |  |
| 1 | 66 (19.8) | 57 (23.1) | 9 (10.3) |  |
| 2 | 162 (48.5) | 122 (49.4) | 40 (46.0) |  |
| 3 | 86 (25.7) | 50 (20.2) | 36 (41.4) |  |
| Pathologic Response |  |  |  | **0.001** |
| Favorable PR (Score 0+1) | 86 (25.7) | 75 (30.4) | 11 (12.6) |  |
| Unfavorable PR (Score 2+3) | 248 (74.3) | 172 (69.6) | 76 (87.4) |  |

IQR, interquartile range.

**Table S2.** Univariable and Multivariable logistic regression in predicting pathologic response (score 0 &1) in overall cohort (N=334).

|  | **Univariable Logistic Regression** | |  |  | **Multivariable Logistic Regression** | |  |
| --- | --- | --- | --- | --- | --- | --- | --- |
|  | **OR** | **95% CI** | ***P* Value** |  | **OR** | **95% CI** | ***P* Value** |
| Age ≥ 65 (years) | 1.34 | [0.82 - 2.18] | 0.248 |  |  |  |  |
| Gender (Male) | 0.74 | [0.45 - 1.22] | 0.236 |  |  |  |  |
| Race (White) | 0.41 | [0.18 - 0.90] | **0.026** |  | 0.44 | [0.19 - 0.99] | **0.048** |
| ASA ≥ 3 | 0.78 | [0.39 - 1.54] | 0.479 |  |  |  |  |
| Pre-NAT Stage |  |  |  |  |  |  |  |
| BR/LAPC | 0.49 | [0.24 - 1.03] | **0.059** |  | 0.98 | [0.41 - 2.32] | 0.954 |
| Resectable | 1 [REF] |  |  |  |  |  |  |
| Type of Neoadjuvant |  |  |  |  |  |  |  |
| 5-FU based | 0.85 | [0.48 - 1.49] | 0.575 |  |  |  |  |
| Gem-based | 1 [REF] |  |  |  |  |  |  |
| Duration of NAT ≥ 4 months | 0.66 | [0.39 - 1.12] | 0.126 |  |  |  |  |
| Chemo-Radiation (vs. Chemotherapy only) | 0.36 | [0.19 - 0.66] | **<0.001** |  | 0.39 | [0.19 - 0.79] | **0.008** |
| Radiation type (SBRT) | 1.16 | [0.55 -2.43] | 0.696 |  |  |  |  |
| Baseline Ca19-9 ≥200 | 1.08 | [0.61 - 1.89] | 0.795 |  |  |  |  |
| Tumor location (Head/neck) | 0.70 | [0.39 - 1.26] | 0.237 |  |  |  |  |
| Grade (Poor) | 1.02 | [0.57 - 1.83] | 0.946 |  |  |  |  |
| OSA | 3.01 | [1.51 - 5.99] | **0.002** |  | 2.72 | [1.35 - 5.49] | **0.005** |
| OR, odds ratio; CI, confidence interval. | |  |  |  |  |  |  |

**Table S3.** Univariable and Multivariable Cox regression in predicting overall survival in overall cohort (N=334).

|  | **Overall Survival (OS)** | | | | | |
| --- | --- | --- | --- | --- | --- | --- |
|  | **Univariable** | |  | **Multivariable** | |  |
|  | **Cox Regression** | |  | **Cox Regression** | |  |
|  | **HR** | **95% CI** | ***P*** | **HR** | **95% CI** | ***P*** |
| Age ≥ 65 years | 1.29 | [0.93 - 1.79] | 0.122 |  |  |  |
| Gender (Male) | 1.16 | [0.84 - 1.61] | 0.349 |  |  |  |
| Race (White) | 0.72 | [0.48 - 1.09] | 0.118 |  |  |  |
| ASA ≥ 3 | 1.39 | [0.89 - 2.18] | 0.145 |  |  |  |
| Pre-NAT Stage |  |  |  |  |  |  |
| BR/LAPC | 1.1 | [0.71 - 1.62] | 0.752 |  |  |  |
| Resectable | 1 [REF] | |  |  |  |  |
| Type of Neoadjuvant |  |  |  |  |  |  |
| 5-FU-Based | 0.88 | [0.62 - 1.24] | 0.452 |  |  |  |
| Gem-Based | 1 [Ref] |  |  |  |  |  |
| Duration of NAT ≥ 4 months | 0.81 | [0.57 - 1.14] | 0.220 |  |  |  |
| Chemo-Radiation | 1.31 | [0.92 - 1.88] | 0.107 |  |  |  |
| Chemotherapy Alone | 1 [Ref] |  |  |  |  |  |
| Baseline Ca19-9 ≥200 | 1.34 | [0.96 - 1.86] | 0.085 | 1.11 | [0.79 - 1.57] | 0.541 |
| Grade (Poor) | 1.58 | [1.13 - 2.22] | **0.008** | 1.60 | [1.12 - 2.29] | **0.010** |
| T-stage ≥ T3 | 1.62 | [1.17 - 2.24] | **0.004** | 1.11 | [0.77 - 1.60] | 0.574 |
| N-stage ≥ N1 | 2.15 | [1.55 - 2.97] | **<0.001** | 1.89 | [1.27 - 2.80] | **0.002** |
| Lymphovascular Invasion | 2.34 | [1.68 - 3.26] | **<0.001** | 1.53 | [1.05 - 2.26] | **0.028** |
| Perineural Invasion | 2.24 | [1.55 - 3.25] | **<0.001** | 1.33 | [0.86 - 2.05] | 0.208 |
| Margin Class (R1) | 1.33 | [0.82 - 2.16] | 0.242 |  |  |  |
| OSA | 1.58 | [1.12 - 2.24] | **0.009** | 1.24 | [0.86 - 1.78] | 0.249 |
| Favorable PR (score 0/1) | 0.57 | [0.39 - 0.89] | **0.010** | 0.64 | [0.42 – 0.97] | **0.036** |
| Adjuvant Chemotherapy | 0.72 | [0.52 - 0.99] | **0.045** | 0.57 | [0.39 - 0.81] | **0.002** |
| HR, hazard ratio; CI, confidence interval. | | |  |  |  |  |

**Table S4.** Univariable and Multivariable Cox regression in predicting recurrence-free survival in overall cohort (N=334).

|  | **Recurrence-Free Survival (RFS)** | | | | | |
| --- | --- | --- | --- | --- | --- | --- |
|  | **Univariable** | |  | **Multivariable** | |  |
|  | **Cox Regression** | |  | **Cox Regression** | |  |
|  | **HR** | **95% CI** | ***P*** | **HR** | **95% CI** | ***P*** |
| Age ≥ 65 years | 0.89 | [0.67 - 1.18] | 0.417 |  |  |  |
| Gender (Male) | 0.97 | [0.73 - 1.28] | 0.966 |  |  |  |
| Race (White) | 0.65 | [0.46 - 0.92] | **0.016** | 0.65 | [0.44 - 0.94] | **0.022** |
| ASA ≥ 3 | 1.16 | [0.80 - 1.66] | 0.432 |  |  |  |
| Pre-neoadjuvant Stage |  |  |  |  |  |  |
| BR/LAPC | 1.07 | [0.74 - 1.53] | 0.727 |  |  |  |
| Resectable | 1 [REF] | |  |  |  |  |
| NAT Regimen |  |  |  |  |  |  |
| 5-FU-Based | 0.88 | [0.65 - 1.19] | 0.412 |  |  |  |
| Gem-Based | 1 [Ref] |  |  |  |  |  |
| Duration of NAT ≥ 4 months | 1.16 | [0.86 - 1.57] | 0.326 |  |  |  |
| Chemo-Radiation | 1.12 | [0.83 - 1.52] | 0.443 |  |  |  |
| Chemotherapy Alone | 1 [Ref] |  |  |  |  |  |
| Baseline Ca19-9 ≥200 | 1.41 | [1.05 - 1.91] | **0.024** | 1.25 | [0.92 - 1.70] | 0.160 |
| Grade (Poor) | 1.43 | [1.06 - 1.93] | **0.020** | 1.21 | [0.87 - 1.69] | 0.250 |
| T-stage ≥ T3 | 1.55 | [1.17 - 2.07] | **0.003** | 1.23 | [0.89 - 1.71] | 0.208 |
| N-stage ≥ N1 | 2.08 | [1.56 - 2.76] | **<0.001** | 1.54 | [1.10 - 2.16] | **0.012** |
| Lymphovascular Invasion | 1.59 | [1.18 - 2.13] | **0.002** | 1.19 | [0.83 - 1.71] | 0.340 |
| Perineural Invasion | 1.62 | [1.20 - 2.19] | **0.002** | 0.96 | [0.66 - 1.41] | 0.848 |
| Margin Class (R1) | 1.23 | [0.79 - 1.91] | 0.345 |  |  |  |
| OSA | 1.32 | [0.97 - 1.81] | 0.080 | 1.10 | [0.78 - 1.55] | 0.591 |
| Favorable PR (score 0/1) | 0.52 | [0.36 - 0.75] | **<0.001** | 0.77 | [0.49 - 1.19] | 0.247 |
| Adjuvant Chemotherapy | 107 | [0.79 - 1.43] | 0.653 |  |  |  |
| HR, hazard ratio; CI, confidence interval. | | |  |  |  |  |

**Figure S1**: Kaplan Meier survival curves comparing superior recurrence-free survival between OSA and non-OSA patients (A), and between favorable PR (score 0/1) and unfavorable PR (score 2/3). OSA, obstructive sleep apnea; non-OSA, without obstructive sleep apnea; PR, pathologic response.


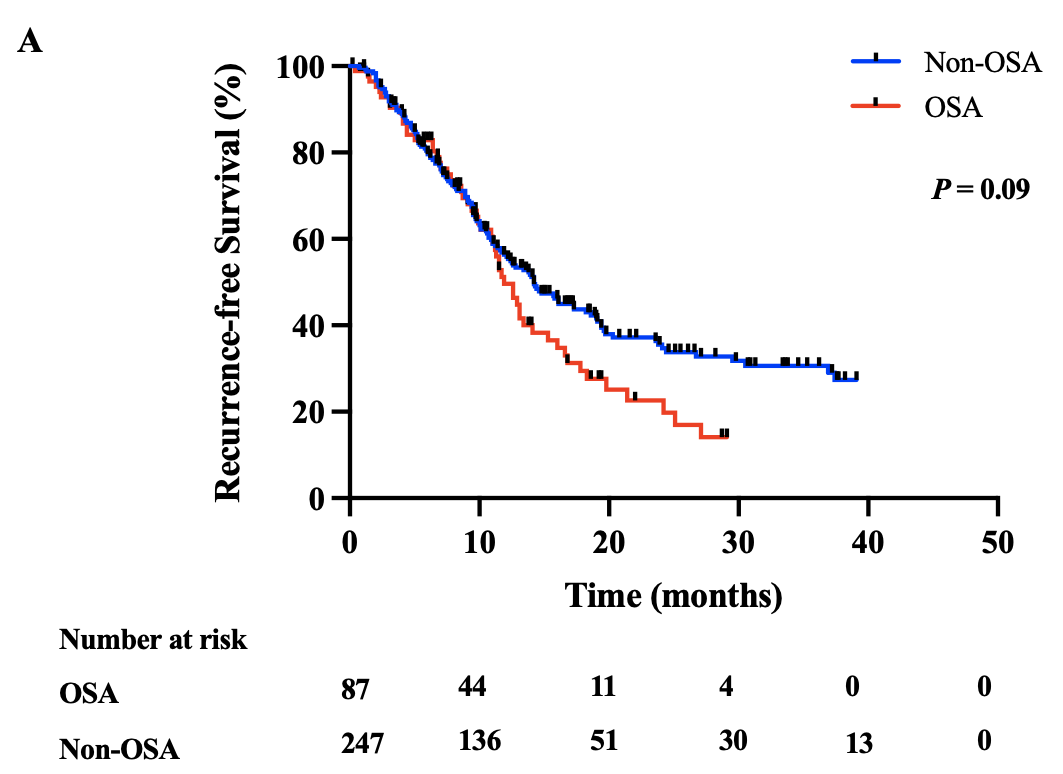


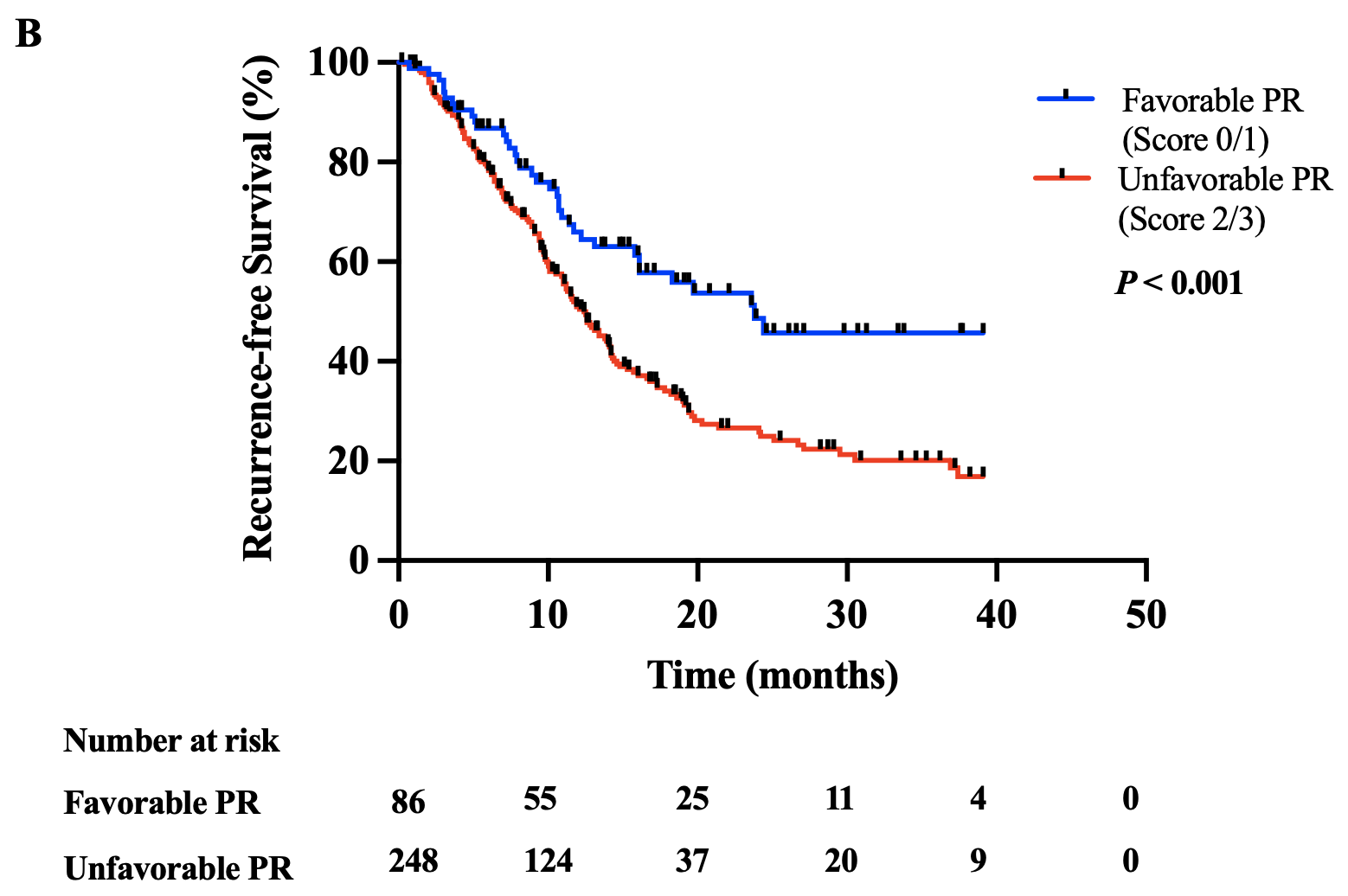

Supplement: Supplementary file 1 — Supporting Information [file MCO2-3-e184-s001.docx]
